# Functional outcomes in ICU – what should we be using? - an observational study

**DOI:** 10.1186/s13054-015-0829-5

**Published:** 2015-03-29

**Authors:** Selina M Parry, Linda Denehy, Lisa J Beach, Sue Berney, Hannah C Williamson, Catherine L Granger

**Affiliations:** Department of Physiotherapy, The University of Melbourne, Level 7 Alan Gilbert Building, 161 Barry St, Parkville, 3010 Melbourne, VIC Australia; Department of Physiotherapy, Melbourne Health, 3050 Melbourne, VIC Australia; Institute for Breathing and Sleep, 3084 Melbourne, VIC Australia; Department of Physiotherapy, Austin Health, 3084 Melbourne, VIC Australia

## Abstract

**Introduction:**

With growing awareness of the importance of rehabilitation, new measures are being developed specifically for use in the intensive care unit (ICU). There are currently 26 measures reported to assess function in ICU survivors. The Physical Function in Intensive care Test scored (PFIT-s) has established clinimetric properties. It is unknown how other functional measures perform in comparison to the PFIT-s or which functional measure may be the most clinically applicable for use within the ICU. The aims of this study were to determine (1) the criterion validity of the Functional Status Score for the ICU (FSS-ICU), ICU Mobility Scale (IMS) and Short Physical Performance Battery (SPPB) against the PFIT-s; (2) the construct validity of these tests against muscle strength; (3) predictive utility of these tests to predict discharge to home; and (4) the clinical applicability. This was a nested study within an ongoing controlled study and an observational study.

**Methods:**

Sixty-six individuals were assessed at awakening and ICU discharge. Measures included: PFIT-s, FSS-ICU, IMS and SPPB. Bivariate relationships (Spearman’s rank correlation coefficient) and predictive validity (logistic regression) were determined. Responsiveness (effect sizes); floor and ceiling effects; and minimal important differences were calculated.

**Results:**

Mean ± SD PFIT-s at awakening was 4.7 ± 2.3 out of 10. On awakening a large positive relationship existed between PFIT-s and the other functional measures: FSS-ICU (rho = 0.87, p < 0.005), IMS (rho = 0.81, p < 0.005) and SPPB (rho = 0.70, p < 0.005). The PFIT-s had excellent construct validity (rho = 0.8, p < 0.005) and FSS-ICU (rho = 0.69, p < 0.005) and IMS (rho = 0.57, p < 0.005) had moderate construct validity with muscle strength. The PFIT-s and FSS-ICU had small floor/ceiling effects <11% at awakening and ICU discharge. The SPPB had a large floor effect at awakening (78%) and ICU discharge (56%). All tests demonstrated responsiveness; however highest effect size was seen in the PFIT-s (Cohen’s d = 0.71).

**Conclusions:**

There is high criterion validity for other functional measures against the PFIT-s. The PFIT-s and FSS-ICU are promising functional measures and are recommended to measure function within the ICU.

**Trial registration:**

Clinicaltrials.gov NCT02214823. Registered 7 August 2014).

**Electronic supplementary material:**

The online version of this article (doi:10.1186/s13054-015-0829-5) contains supplementary material, which is available to authorized users.

## Introduction

Impairment in physical function is a significant problem for survivors of critical illness [1-3]. Physical function refers to ‘the ability to carry out various activities that require physical capability ranging from self-care to more vigorous activities that require increasing degrees of mobility, strength or enduranceʼ [4]. The International Classification of Functioning (ICF) framework provides a conceptual model to guide patient assessment, which includes examination of impairment, activity limitations and participation restrictions [2,5]. In survivors of critical illness, measurement of physical function using performance-based tests provides information on the patients’ activity limitations. There are currently 26 self-report and performance-based measures reported in the literature to assess physical function in ICU survivors [6].

When selecting the most appropriate outcome measure to assess physical function, clinicians and researchers need to consider which outcome measures have robust clinimetric properties [2]. This includes the ability of an outcome measure to measure what it is intended to measure, that is, how well the test results relate to data obtained from the gold standard instrument (criterion-concurrent validity); how well the outcome obtains data, as hypothesized, when compared to an instrument measuring a similar construct (convergent or construct validity); or how well data predict an outcome (predictive validity/utility) [7,8]. Additionally, the clinical applicability of the outcome measure is also important. This includes whether there is a floor or ceiling effect; the ability of the outcome measure to detect meaningful change over time (responsiveness) [8]; and whether there is a known minimal important difference (the smallest change in the outcome measure that patients and clinicians consider to be clinically relevant) [9]. These clinimetric properties should be examined specifically within the setting in which the outcome measure will be used [10]. This is particularly important for a challenging environment such as ICU, where fluctuations in patient mental alertness, ability to follow commands, and both rapid changes in medical stability and a confined space may impact on the choice, reliability and validity of outcome measures [2,11,12].

Whilst 26 different functional measures have been described for use within critically ill patients, there are currently only six published functional measures that have been developed specifically for the ICU setting and have undergone clinimetric evaluation [6]. These measures are the Physical Function in Intensive care Test scored (PFIT-s) [13], Chelsea Critical Care Physical Assessment tool (CPAx) [14], Perme mobility scale [15], Surgical intensive care unit Optimal Mobilization Score (SOMS) [16], ICU Mobility Scale (IMS) [17], and the Functional Status Score for the ICU (FSS-ICU) [18,19]. To date, studies of clinimetric properties have been primarily limited to a single component, such as reliability testing [6]. There is also growing interest in the application of the Short Physical Performance Battery (SPPB) [20] or its components [21] to evaluate functional recovery in individuals with critical illness. The SPPB is a battery tool, originally developed for use in the geriatric population [22,23] and may discriminate between individuals with critical illness who receive early rehabilitation versus usual care [20]. However, there have been no published studies on the SPPB examining specifically the clinimetric properties of this test within the ICU setting.

The clinimetric properties of functional measures for use within the ICU setting are still in early development. The PFIT-s [13,24] and CPAx tools [14,25] have the most established clinimetric properties in terms of reliability, validity and responsiveness. The PFIT-s can be used to guide exercise prescription within the ICU as well as measuring functional recovery [13,26]. The PFIT-s can be used as a reference standard against which other functional measures can be compared.

Three functional measures, which were selected for comparison against the PFIT-s within this study, were: FSS-ICU, IMS and the SPPB. It is unknown how these other functional measures perform in comparison to the PFIT-s or which of these four functional measures may be the most clinically applicable for use within the ICU. Therefore, the aims of this study were to determine (1) the criterion validity of the FSS-ICU, IMS and SPPB against the PFIT-s; (2) the construct validity of these outcome measures against measurement of muscle strength; (3) the predictive utility of these outcome measures to predict patients who would discharge directly home from hospital; and (4) the clinical applicability of the outcome measures (floor and ceiling effects, responsiveness and minimal important difference). It was hypothesized that the FSS-ICU would have the strongest positive correlation with the PFIT-s (correlation >0.50). The COnsensus-based Standards for the selection of health status Measurement INstruments (COSMIN) guidelines [27] and the Strengthening the Reporting of Observational Studies in Epidemiology (STROBE) guidelines [28] were followed in reporting this study.

## Materials and methods

### Study design, setting and participants

This was a nested observational study within an ongoing controlled trial (NCT02214823) and an observational study conducted in major mixed (medical and surgical) ICUs in Melbourne, Australia. Consecutive participants were recruited between July 2012 and June 2014 for the two main trials. Both sites had institutional ethical approval (Melbourne Health and Austin Health Human Research Ethics Committees) and participants provided written informed consent. Participants were included in the primary trials if they were admitted to an ICU, were more than 18 years old, English speaking, and were mechanically ventilated for 48 hours and expected to remain in the unit for at least four days. Patients were excluded from the primary trials if they had a premorbid physical or cognitive impairment that would prevent exercise, or were admitted with a new neurological insult such as stroke or spinal cord injury. All patients were required to be able to ambulate at least 10-metres independently prior to ICU admission (+/− gait aid). Patients were included in the nested study as a convenience sample if they had been assessed at both awakening and ICU discharge.

### Procedure

Assessments were performed twice in the ICU for each patient: on awakening and ICU discharge. Day of awakening was defined as when the patient scored greater than three out of five on the De Jonghe comprehension criteria on two consecutive occasions within a 6-hour period [29]. Between the two time points of testing (awakening and ICU discharge), patients received usual-care rehabilitation involving early mobility activities such as active exercises in bed, sitting on the edge of the bed, standing, marching, and walking. At each time point of testing, the participants completed the PFIT-s and a range of additional functional tests (FSS-ICU, IMS, SPPB). The Medical Research Council-Sum Score (MRC-SS) [30] was assessed at awakening in order to determine the incidence of ICU-acquired weakness (ICU-AW) within the population studied. The order of testing varied, and was not controlled within this study. In order to minimize fatigue as an issue, all tests were completed within a 12-hour period, and participants were required to return to baseline based on clinical parameters such as heart rate, respiratory rate and oxygen saturation levels before the next functional test was undertaken. Patients were stable and unchanged in the time between completing the PFIT-s and additional functional tests. All assessors were qualified physical therapists and had received standardized training in the outcome measures (PFIT-s, SPPB, FSS-ICU, IMS, MRC-SS) from one senior physical therapist. The same assessor (where possible) performed awakening and ICU discharge measures within the same patient.

### Physical function in intensive care test scored

The PFIT-s is a battery outcome measure involving four components: sit to stand assistance, marching on the spot cadence, shoulder flexor and knee extensor strength. The PFIT-s scores range from 0 (able to perform strength testing only with a maximum score of 2 out of 5 for shoulder and knee) to 10 (performance without any difficulty) [13,31]. This tool has established validity, reliability and a minimal clinically important difference (MCID) of 1.5 points out of 10 [13,31].

### Additional outcome measures

Participants also completed three additional functional measures: the FSS-ICU, IMS and SPPB. The FSS-ICU involves assessment of five functional tasks (rolling, supine to sit transfers, unsupported sitting, sit to stand transfers, and ambulation). The five tasks are scored on a seven-point scoring system from the Functional Independence Measure [18,19]. Higher scores represent better function and the total score ranges from 0 to 35. The FSS-ICU was originally developed for use within the ICU setting [18] however there has been no evaluation of the clinimetric properties of the FSS-ICU specifically within the ICU setting. Despite this the FSS-ICU has been shown to be responsive to change over time and a valid predictor of discharge destination when implemented in a long-term acute care facility [19].

The IMS is an 11-item categorical scale that rates the patient's highest level of mobility, where 0 = nothing and 10 = walking independently without a gait aid [17]. Although there is high inter-rater reliability for this measure in the ICU setting, there are no published data on other clinimetric properties (validity, responsiveness) [17].

The SPPB is a battery tool, which was originally developed for use in the geriatric population [22,23]. A score of 0 to 12 (higher scores indicating better function) is based on the performance of three tasks: gait speed, chair rise time (five times sit to stand) and standing balance (tandem, semi-tandem and side by side) [22]. This is increasingly being used in the ICU setting and to date there are no published data on the clinimetric properties or clinical applicability of the SPPB within the ICU setting.

Baseline demographics were recorded, including age, sex, body mass index, admission diagnosis and severity of illness (Acute physiological and chronic health evaluation (APACHE) II within first 24 hours of ICU admission). Additionally ICU length of stay (LOS), mechanical ventilation (MV) duration in days, and acute hospital discharge destination were recorded.

### Sample size

The sample size was 66. Sample sizes of ≥50 participants are recommended for studies assessing clinimetric properties of measurements to enhance the generalizability of findings [32,33]. The examination of the SPPB was performed in a subgroup (n = 23) within this nested study. Only participants from one of the two trials completed the SPPB. Therefore SPPB analyses are underpowered and results should be viewed with caution. The measurement of SPPB was opportunistic to enable preliminary examination of the clinimetric properties of the SPPB in individuals with critical illness, which had not been reported within the literature to date.

### Statistical analyses

Data were analysed with SPSS Windows Version 22.0 (SPSS, Chicago, IL, USA). Data were assessed for normality using the Kolmogorov-Smirnov statistic. Parametric data are presented as mean and SD, and non-parametric data are presented as median and IQR. Spearman’s rank correlation coefficient was used to assess the bivariate relationships between test scores (PFIT-s, FSS-ICU, IMS and SPPB) [7]. Coefficients were interpreted as: little (0.00 to 0.25), fair (0.25 to 0.50), moderate (0.50 to 0.75) and excellent association (0.75 to 1.0) [8]. Alpha was set at 0.05 for all analyses.

Predictive utility of the tests were assessed using logistic regression analyses to investigate the ability of the test to predict likelihood of discharge directly to home compared to other destinations. Logistic regression analyses were run separately for the PFIT-s, FSS-ICU and IMS on awakening and ICU discharge (SPPB was not assessed with logistic regression as this had no bivariate correlation with discharge destination). The test score on awakening was the variable of interest (independent variable) and was included in all regression models. The outcome of interest (dependent variable) was the dichotomous variable of discharge directly to home and was coded as yes (discharged directly home) or no (not discharged directly home). Potential covariates were: age (in years), sex (coded as male or female), body mass index (in kg/m^2^), APACHE II (in points), MV duration (in days), MRC-SS on awakening (in points), and ICU LOS (in days). The potential covariates with significant bivariate correlation with the dependent variable were included in the model if collinearity was not identified. Collinearity was assessed using Spearman’s rank correlation coefficient and defined as rho ≥0.7. Overfitting of the model was avoided with no more than three independent variables included in the final model. Conformity to a linear gradient for variables was examined with inspection of a plot with locally weighted scatterplot smoothing (LOWESS). Goodness of fit was examined using the Hosmer-Lemeshow goodness of fit test and poor fit was defined as alpha <0.05. In addition the differences in test scores on awakening of those participants discharged directly home versus those discharged to other destinations were determined using the independent *t*-test for continuous parametric data, the Mann-Whitney *U*-test for ordinal non-parametric data and the Chi-square test for categorical data.

Floor and ceiling effects of the PFIT-s, FSS-ICU, IMS and SPPB were determined using the percentage of occasions when participants scored the lowest or highest score possible for the test. Change over time, from awakening to ICU discharge, was assessed using the paired *t*-test for parametric data [8] and the Wilcoxon signed rank test for non-parametric data [8]. Responsiveness of each test was determined with calculation of the effect size. For parametric data this was defined as Cohen’s *d* and calculated as the mean difference divided by pooled SD [34]. For non-parametric data this was defined as *r* = *Z* divided by the square root of the sample size [8,34]. Thresholds for interpretation of the change were: small (0.2 to 0.49), moderate (0.5 to 0.79) and large (≥0.8) [34,35].

The minimal important differences (MIDs) of the continuous and ordinal tests were determined using distribution-based estimation with calculation of the standard error of the measurement (SEM) and effect size (ES). The SEM was calculated as σ_1_√(1-r), where σ_1_ was the baseline SD of the test score and *r* was the test-retest reliability coefficient of the test [36]. A moderate ES is considered a clinically important effect and was calculated using the formula 0.5 × SD of the change scores [37].

## Results

The PFIT-s and additional functional tests were conducted with 66 patients. The characteristics of the cohort studied are reported in Table 1. All participants were previously independent and prior to hospitalization in the ICU were from home. The mean ± SD PFIT-s on awakening was 4.7 ± 2.3 out of 10 (Table 1).

**Table 1:** **Demographics of the cohort**

**Variable**	**Total sample (n = 66)**
Male, n (%)	40 (61%)
Age, mean ± SD	58 ± 17
Body mass index, kg/m^2^ median (IQR)	28 (24 to 32)
Acute physiological and chronic health evaluation II, score^a^, mean ± SD	21 ± 7
ICU admission diagnosis	
- respiratory, n (%)	14 (21%)
- gastrointestinal, n (%)	12 (18%)
- sepsis, non-pulmonary, n (%)	13 (20%)
- cardiac, n (%)	18 (27%)
- trauma, n (%)	5 (8%)
- other, n (%)	4 (6%)
Medical Research Council (MRC) sum-score on awakening median (IQR)	48 (39 to 54)
ICU-acquired weakness (<48/60 MRC) on awakening	28 (42%)
ICU length of stay, days, median (IQR)	8 (5 to 15)
Mechanical ventilation time, days, median(IQR)	5 (3 to 10)
Acute hospital discharge destination	
- home, n (%)	37 (56%)
- inpatient rehabilitation facility, n (%)	20 (30%)
- deceased in hospital, n (%)	3 (4%)
- other or unknown, n (%)	6 (9%)
Physical function in ICU test scored, on awakening, mean ± SD	4.7 ± 2.3
Physical function in ICU test scored, at ICU discharge, mean ± SD	6.3 ± 2.2
Functional status score for ICU, on awakening, median (IQR)	12.0 (6.0 to 17.0)
Functional status score for ICU, at ICU discharge, median (IQR)	17.0 (12.0 to 26.5)
ICU mobility scale, on awakening, median (IQR)	3.5 (1.0 to 5.0)
ICU mobility scale, at ICU discharge, median (IQR)	6.0 (5.0 to 8.0)
Short Physical Performance Battery, on awakening, median (IQR) (n = 23)	0.0 (0.0 to 0.0)
Short Physical Performance Battery, at ICU discharge, median (IQR) (n = 23)	0.0 (0.0 to 5.0)

^a^APACHE II score was determined within the first 24 hours of ICU admission.

### Validity

**Figure 1:**
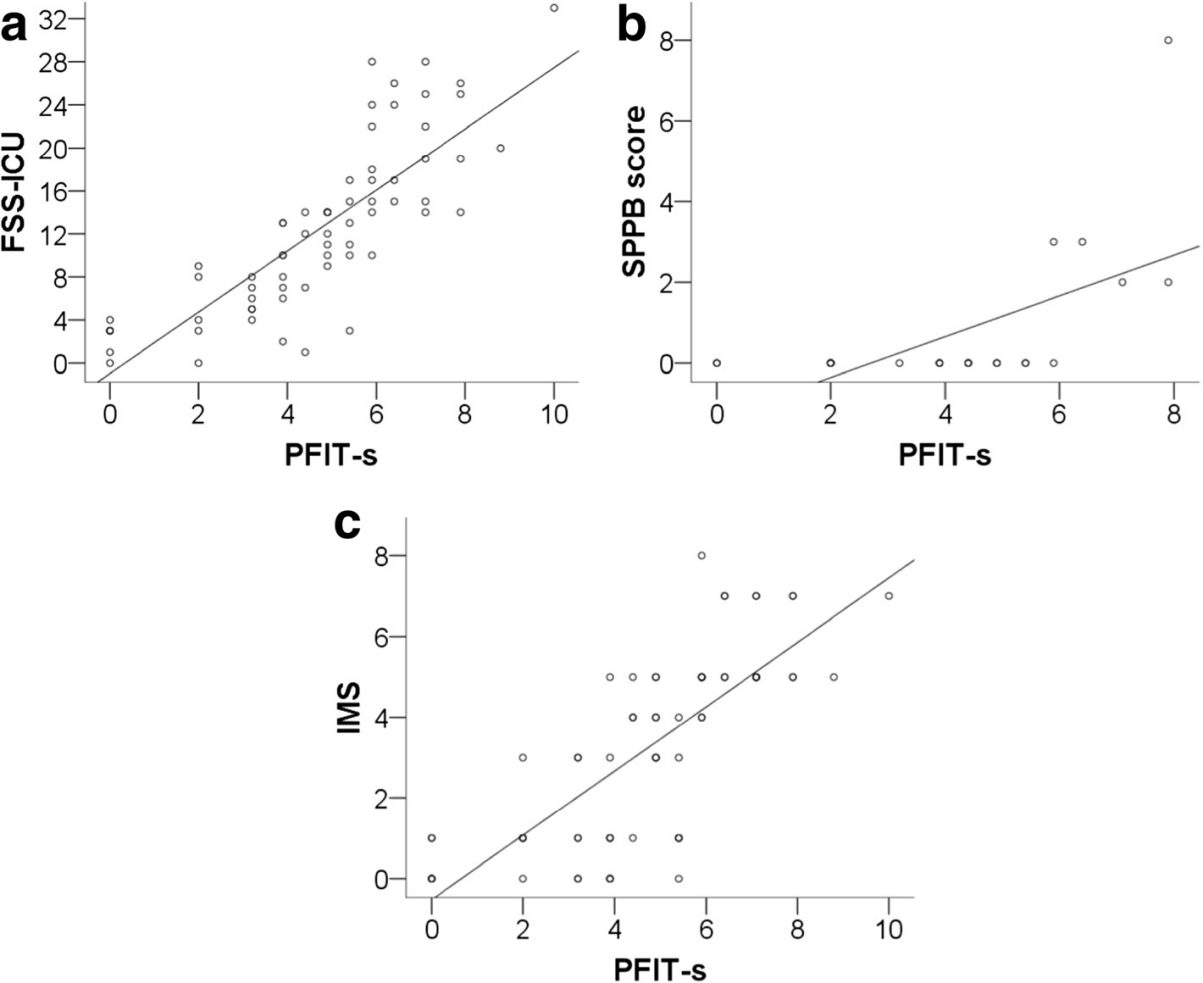
**Relationship between physical function in intensive care test scored (PFIT-s) and (a) Functional Status Score for the Intensive Care Unit (FSS-ICU), (b) Short Physical Performance Battery (SPPB) and (c) ICU mobility scale (IMS) on awakening.**

There was moderate to large criterion validity between the PFIT-s and the three other functional tests (Figure 1). On awakening large positive relationships existed between PFIT-s and the FSS-ICU (n = 66, rho = 0.87, 95% CI = 0.79, 0.92, *P* <0.005) (Figure 1a) and IMS (n = 66, rho = 0.81, 95% CI = 0.70, 0.88, P <0.005) (Figure 1c); and a moderate positive relationship existed between the PFIT-s and SPPB (n = 23, rho = 0.70, 95% CI = 0.47, 0.83, *P* <0.005) (Figure 1b). At ICU discharge large positive relationships existed between PFIT-s and FSS-ICU (n = 66, rho = 0.85, 95% CI = 0.77, 0.90, *P* <0.005) and SPPB (n = 23, rho = 0.86, 95% CI = 0.73, 0.91, *P* <0.005); and a moderate positive relationship existed between the PFIT-s and IMS (n = 64, rho = 0.66, 95% CI = 0.49, 0.80, *P* <0.005).

The PFIT-s demonstrated excellent construct validity with measurement of muscle strength: on awakening a large positive relationship existed between the PFIT-s and MRC sum-score (n = 66, rho = 0.8, 95% CI = 0.68, 0.89, *P* <0.005). In addition, moderate positive relationships existed between FSS-ICU and MRC sum-score (n = 66, rho = 0.69, 95% CI = 0.50, 0.83, *P* <0.005), and between IMS and MRC sum-score (n = 66, rho = 0.57, 95% CI = 0.36, 0.74, *P* <0.005). There was no relationship between SPPB and MRC sum-score on awakening (n = 23, rho = 0.30, 95% CI = −0.08, 0.62, *P* = 0.161).

### Predictive utility

Fifty-six percent (n = 37) of participants were discharged directly home from their acute hospital stay (Table 1). Higher PFIT-s scores on awakening (odds ratio (OR) = 1.59, *P* = 0.004) and lower age were significant factors in determining whether the patient was discharged directly home (Additional file 1: Table S1); this was not found for FSS-ICU (*P* = 0.642) or IMS (*P* = 0.143) when assessed on awakening. Lower age and higher test scores at ICU discharge were significant factors in determining discharge to home: PFIT-s (OR = 1.56, *P* = 0.005), FSS-ICU (OR = 1.09, *P* = 0.013) and IMS (OR = 1.54, *P* = 0.011) (Additional file 1: Table S1). There was a significant difference in the PFIT-s, IMS and FSS-ICU scores on awakening between participants who were discharged home from the acute hospital versus those who were discharged to another location (*P* <0.05), this was not the case for the SPPB (*P* >0.05).

### Clinical applicability

Table 2 provides the floor and ceiling effects for each test, as well as the range of test scores at both awakening and ICU discharge.

**Table 2:** **Floor and ceiling effects**

**Test**	**Floor effect n/total n assessed (%)**	**Ceiling effect n/total n assessed (%)**	**Range of test scores at each time point**
PFIT-s on awakening	6/66 (9.1%)	1/66 (1.5%)	0 to 10 out of 10
PFIT-s at ICU discharge	1/66 (1.5%)	7/66 (10.6%)	0 to 10 out of 10
FSS-ICU on awakening	2/66 (3.0%)	0/66 (0%)	0 to 33 out of 35
FSS-ICU at ICU discharge	0/66 (0%)	2/66 (3.0%)	2 to 35 out of 35
IMS on awakening	11/66 (16.7%)	0/66 (0%)	0 to 8 out of 10
IMS at ICU discharge	0/64 (0%)	3/64 (4.7%)	1 to 10 out of 10
SPPB on awakening	18/23 (82.6%)	0/23 (0%)	0 to 8 out of 12
SPPB at ICU discharge	13/23 (56.5%)	0/23 (0%)	0 to 11 out of 12

FSS-ICU, Functional Status Score for the Intensive Care Unit; IMS, ICU mobility scale; n, number; PFIT-s, Physical Function in Intensive Care Test scored; SPPB, Short Physical Performance Battery.

There were significant improvements from awakening to ICU discharge in the PFIT-s (mean difference = 1.59, 95% CI = 1.12, 2.06, *P* <0.005), FSS-ICU (*Z* = −5.34, *P* <0.005), IMS (*Z* = −6.71, P <0 0.005) and SPPB (Z = −2.23, p = 0.026). All tests demonstrated responsiveness to change, however the highest effect size was seen for the PFIT-s: The effect sizes of the PFIT-s and IMS were 0.71 and 0.59 respectively, which represent a moderate responsiveness to change. The effect sizes of the FSS-ICU and SPPB were 0.46 and 0.33 respectively, which represent small responsiveness to change.

The minimal important differences for the three tests were estimated to be between 1.0 to 1.4 points for the PFIT-s, 4.3 to 5.6 points for the FSS-ICU, and 1.5 to 1.3 points for the SPPB according to calculation of the ES and SEM respectively.

## Discussion

Physical function is an important outcome to measure in survivors of critical illness. In a moderately unwell cohort of ICU survivors from two mixed medical/surgical ICUs we found that there was high criterion validity for other functional measures against the PFIT-s especially for the FSS-ICU tool. The PFIT-s had strong construct validity with measurement of muscle strength, and both the FSS-ICU and IMS had moderate construct validity. There was no relationship between SPPB and muscle strength at awakening or ICU discharge. All functional measures except the SPPB were able to discriminate and predict future discharge destination.

The PFIT-s had high construct validity with muscle strength and was predictive of discharge destination when measured on awakening or at ICU discharge. This is in consensus with what has previously been reported within the ICU literature [13,31]. The MID for the PFIT-s was estimated to be between 1.0 to 1.4 points in this study, which was similar to that reported by Denehy and colleagues in 2013 (1.5 points out of 10.0) [13] adding further validity to the cutoff point previously developed. The floor and ceiling effects for the PFIT-s were small in our study (9 and 11% respectively). This is in contrast to previous studies, which reported a floor effect of 22 to 32% on awakening and a ceiling effect of 5 to 22% respectively [13,31]. The differences may in part be due to differences in patient cohort demographics and sedation practices and provisions of therapy within the units. In comparison to the study by Nordon-Craft and colleagues [31] there was a marked difference in overall MV duration and ICU LOS compared to our study (median MV duration of 12 days and ICU LOS of 20 days versus MV duration of 5 days and ICU LOS of 8 days). There was a large floor effect observed at baseline by Nordon-Craft and colleagues (32%) [31] compared to our study with a floor effect of 9% on awakening. Similar to the findings of Denehy and colleagues [13] this paper identified that a higher PFIT-s (better function) predicted a greater likelihood of return to home. The demographics within the Australian study [13] were more comparable to the sample examined within our study.

Compared to the PFIT-s the FSS-ICU was the most robust functional outcome measure in the ICU setting. It was predictive of discharge destination when tested at ICU discharge and had small floor/ceiling effects within the ICU setting. Floor and ceiling effects are of concern for longitudinal studies as they limit the ability to detect change over time in terms of improvement and/or deterioration in functional recovery [7]. The floor effect observed at awakening was less than 15% (the acceptable cutoff for outcome measures) [38] for the PFIT-s and FSS-ICU (<10%). In contrast there was a large floor effect for SPPB on awakening and ICU discharge time points and a moderate floor effect at ICU awakening for IMS (17%).

The SPPB is a high-level physical function outcome as it examines gait speed, balance control and sit-to-stand repetitions. Although power was not achieved for the SPPB outcome, large floor effects were observed for the SPPB suggesting it may not be a feasible measure for use within the ICU setting. Floor and ceiling effects are influenced by patient characteristics and therefore differences in sample characteristics may affect the choice of test used to evaluate physical function. For example the SPPB contains tasks requiring higher-level functional performance compared to the PFIT-s and FSS-ICU, which may be more sensitive in individuals with greater severity of illness and impairment to be able to detect change over time in the early ICU admission period. It is important that patient characteristics are considered when selecting a test. The SPPB may be more appropriate as a measure in the post ICU setting in the acute hospital wards or post hospital discharge.

The minimal important difference is the smallest change in an outcome that is considered to be clinically relevant [9]. There are two main methods used to determine the minimal important difference, the distribution-based method and the anchor-based method [39]. The distribution-based method utilizes statistical analyses to determine the minimal important difference using the degree of test score variability. The main disadvantage with this method is that it does not take into account whether the clinician or patient feels that change is clinically meaningful [40]. The anchor-based estimation takes into account a patient-related anchor such as the global rating of change scale to determine if the patient is clinically changed [39]. Future studies need to determine whether the cutoff values developed using the distribution-based methods remain stable when applying the anchor-based methods and demonstrate a change that is clinically meaningful.

Higher PFIT-s scores (better function) were shown to be predictive of discharge to home on both awakening and ICU discharge time points. Higher FSS-ICU and IMS scores were predictive of discharge to home only at ICU discharge. The differences in the predictive validity of the functional outcomes at the two time points may relate to differences in the individual constructs measured within each measure. The PFIT-s involves evaluation of peripheral muscle strength (shoulder flexion and knee extension); sit to stand assistance and marching in place cadence. In contrast the IMS and FSS-ICU examine a hierarchical level of dependence/assistance required to perform functional tasks such as bed mobility, sitting, standing and walking. Physical function is a complex entity to measure and can encompass a range of different constructs. Therefore, in the future it is important that within functional tests we consider what the individual constructs of a functional test are able to tell us about a patient’s future trajectory of recovery and the prediction of discharge to home and resumption of family, societal and community roles.

In addition to consideration of the clinimetric properties of outcome measures it is important to consider other aspects of utility such as time to complete the measure, equipment and training required or health professional expertise, and availability of the outcome measure for use in clinical practice. The advantage of the four functional measures examined within this study is that they are readily available, and require little dedicated equipment (for example, for the PFIT-s a stopwatch is required for marching in place cadence, or gait aids/chairs if required for ambulation/sitting out of bed components of all functional tests). All outcome measures take <20 minutes to complete.

Interventions aimed at improving functional recovery may not only minimize or improve physical function but may also affect cognitive processing, and emotional health. Therefore measures that evaluate these aspects also need to be examined across different time points in the trajectory of recovery [2,41]. It is important that there is mapping of outcome measures within the ICF framework to capture impairment, activity limitation and participation restrictions across the continuum of recovery. It is likely in the future that there will be overlap in the functional outcomes that are utilized, which enable sensitive monitoring of functional recovery and determination of the efficacy of interventional strategies.

The ICU environment is a challenging setting in which to conduct research, due to patient heterogeneity, severity of illness, and mortality. To improve the ability to compare findings between research studies, there is an urgent need to adopt a standardized core set of outcome measures. Functional recovery and independence is complex and requires individuals to master multiple facets simultaneously [2,41]. For example independent mobility in the community requires not only muscular strength, but postural control, endurance, cognitive processing to anticipate obstacles and respond to the changing demands of the environment surrounding them [2,41]. It is therefore important that outcome measures are adopted that are setting-specific to ensure improvement and/or deterioration in function are meaningfully encapsulated to capture changes in impairment, activity limitations and participation restrictions. For example, the distance a patient ambulates and level of assistance does not provide you with information on the quality of the patient’s ambulation. We hypothesise that there will not be a single functional outcome that can be utilized across the continuum of recovery post critical illness. It is also important to consider different stages of recovery, as this will vary from patient to patient at different time points. For example, some patients are able to complete a 6MWT = six minute walk test at hospital discharge, while others cannot. This will enable the identification of deficits, which may impact on the ability to discharge home and ultimately resume family and societal roles in individuals with very low levels of function, through to higher-functioning individuals after the insult of initial critical illness.

### Limitations

While combining data from these two studies improved the generalizability of findings, the overall sample size was small. This study was underpowered to examine the clinimetric properties of the SPPB and thus, results should be viewed with caution and interpreted as an overall trend in findings. Whilst reliability of the functional outcomes was not examined specifically within this study, the reliability of the PFIT-s and IMS has been previously reported within individuals with critical illness [17,42]. Currently there are no published data on the reliability of the FSS-ICU and this is an area that needs to be addressed in future research. To our knowledge, the reliability of the SPPB scoring has not been reported for individuals with critical illness; it has been shown to have excellent reliability in the general geriatric population [43]. Results from logistic regression need to be validated in an independent sample, and therefore, results on the ability of the PFIT-s, FSS-ICU and IMS to predict discharge destination must be viewed with caution.

This study only examined functional measures within the ICU setting, and the utility of these outcomes in the post ICU setting warrants further examination. The CPAx, Perme mobility and SOMS scales were not examined within this paper and warrant further testing to determine their utility for measuring functional changes within individuals with critical illness. It is essential that rigorous examination of currently utilized functional measures continue to be undertaken in order to determine the most appropriate outcome/s, which can be utilized across the continuum of patient recovery specifically for individuals with critical illness.

## Conclusions

There is excellent criterion validity for other functional measures (FSS-ICU, IMS and SPPB) against the PFIT-s in the ICU setting. Higher PFIT-s scores on awakening were predictive of discharge directly home. All tests were responsive to change, however, the SPPB and IMS were limited by floor effects when used in the ICU. Based on the findings in this study the PFIT-s and FSS-ICU are promising functional measures and should be considered currently when measuring physical function in the ICU in clinical practice and research.

## Key messages

- Impairment in physical function is a significant problem for survivors of critical illness.
- PFIT-s and FSS-ICU are promising functional measures and should be considered when measuring physical function in the ICU.
- A core set of outcome measures, which map impairment, activity limitations and participation restrictions within the ICF framework need to be developed, which can be utilized across different time points of recovery.

## Additional file

Additional file 1: Table S1. Logistic regression models for prediction of discharge directly home. D/C, discharge; FSS-ICU, Functional Status Score for the Intensive Care Unit; IMS, ICU mobility scale; n, number; PFIT-s, Physical Function in Intensive Care Test scored.

## Abbreviations

APACHE II: Acute physiological and chronic health evaluation 2
COSMIN: COnsensus-based standards for the selection of health status Measurement INstruments
CPAx: Chelsea critical care physical assessment tool
FSS-ICU: Functional status score for the intensive care unit
ICF: International classification of functioning
ICU-AW: intensive care unit-acquired weakness
IMS: ICU mobility scale
LOS: length of stay
MCID: minimal clinically important difference
MID: minimal important difference
MRC: Medical Research Council
MV: Mechanical ventilation
PFIT-s: Physical function in intensive care test scored
SEM: standard error of measurement
SPPB: Short physical performance battery
STROBE: Strengthening the reporting of observational studies in epidemiology
