# Supplementary material for: Functional outcomes in ICU – what should we be using? - an observational study
Source: Crit Care. 2015 Mar 29;19(1):127. doi: 10.1186/s13054-015-0829-5 (PMC4404223; doi:10.1186/s13054-015-0829-5)
Supplement: Additional file 1: Table S1. — Logistic regression models for prediction of discharge directly home. D/C, discharge; FSS-ICU, Functional Status Score for the Intensive Care Unit; IMS, ICU mobility scale; n, number; PFIT-s, Physical Function in Intensive Care Test scored. [file 13054_2015_829_MOESM1_ESM.doc]

**Additional file 1: Table S1: Logistic regression models for prediction of discharge directly home**

| **Variable** | **n** | **B** | **S.E** | **Wald** | **df** | **P** | **Odds Ratio** | **95%CI for odds ratio** |
| --- | --- | --- | --- | --- | --- | --- | --- | --- |
| **Model 1** |  |  |  |  |  |  |  |  |
| PFIT-s on awakening | 66 | -0.47 | 0.16 | 8.21 | 1 | 0.004 | 1.59 | 1.16- 2.17 |
| Age | 66 | 0.04 | 0.02 | 3.45 | 1 | 0.063 | 0.96 | 0.93-1.00 |
| Constant |  | -0.20 | 1.52 | 0.02 | 1 | 0.895 | 1.22 |  |
| **Model 2** |  |  |  |  |  |  |  |  |
| PFIT-s at ICU D/C | 66 | -0.44 | 0.15 | 8.03 | 1 | 0.005 | 1.56 | 1.15-2.08 |
| Age | 66 | 0.05 | 0.12 | 5.52 | 1 | 0.019 | 0.95 | 0.92-0.99 |
| Constant |  | -0.24 | 1.51 | 0.03 | 1 | 0.873 | 1.27 |  |
| **Model 3** |  |  |  |  |  |  |  |  |
| PFIT-s at ICU D/C | 66 | -0.34 | 0.15 | 4.82 | 1 | 0.028 | 1.41 | 1.04-1.89 |
| ICU LOS | 66 | 0.11 | 0.05 | 5.53 | 1 | 0.019 | 0.91 | 0.81-0.98 |
| Constant |  | 0.63 | 1.19 | 0.28 | 1 | 0.598 | 0.53 |  |
| **Model 4** |  |  |  |  |  |  |  |  |
| FSS-ICU at ICU D/C | 66 | -0.08 | 0.03 | 6.13 | 1 | 0.013 | 1.09 | 1.02-1.16 |
| Age | 66 | 0.04 | 0.02 | 4.86 | 1 | 0.027 | 0.96 | 0.93-0.99 |
| Constant |  | -1.22 | 1.40 | 0.76 | 1 | 0.384 | 3.33 |  |
| **Model 5** |  |  |  |  |  |  |  |  |
| FSS-ICU at ICU D/C | 66 | -0.09 | 6.24 | 0.013 | 1 | 0.013 | 1.09 | 1.02-1.16 |
| ICU LOS | 66 | 0.14 | 7.88 | 0.005 | 1 | 0.005 | 0.87 | 0.79-0.96 |
| Constant |  | -0.09 | 0.01 | 0.917 | 1 | 0.917 | 1.09 |  |
| **Model 6** |  |  |  |  |  |  |  |  |
| IMS at ICU D/C | 64 | -0.42 | 0.17 | 6.41 | 1 | 0.011 | 1.54 | 1.10-2.13 |
| Age | 64 | 0.05 | 0.02 | 5.81 | 1 | 0.016 | 0.95 | 0.92-0.99 |
| Constant |  | -0.43 | 1.60 | 0.07 | 1 | 0.789 | 1.54 |  |
| **Model 7** |  |  |  |  |  |  |  |  |
| IMS at ICU D/C | 64 | -0.40 | 0.18 | 5.08 | 1 | 0.024 | 1.49 | 1.05-2.08 |
| ICU LOS | 64 | 0.11 | 0.04 | 6.20 | 1 | 0.013 | 0.89 | 0.82-0.98 |
| Constant |  | 1.02 | 1.26 | 0.65 | 1 | 0.418 | 0.36 |  |

*Abbreviations*: 95%CI, 95 percent confidence intervals; D/C, discharge; FSS-ICU, Functional Status Score for the Intensive Care Unit; ICU, intensive care unit; IMS, ICU mobility scale; n, number; PFIT-s, Physical Function in Intensive Care Test scored.
